# Activation of dopamine receptor D1 promotes osteogenic differentiation and reduces glucocorticoid-induced bone loss by upregulating the ERK1/2 signaling pathway

**DOI:** 10.1186/s10020-022-00453-0

**Published:** 2022-02-21

**Authors:** Jie Zhu, Chengcheng Feng, Weicheng Zhang, Zhidong Wang, Mengdan Zhong, Wenkai Tang, Zhifang Wang, Haiwei Shi, Zhengyu Yin, Jiandong Shi, Yu Huang, Long Xiao, Dechun Geng, Zhirong Wang

**Affiliations:** 1 Department of Orthopedics, Zhangjiagang TCM Hospital Affiliated to Nanjing University of Chinese Medicine, Zhangjiagang, 215600 China; 2 Center Laboratory, Zhangjiagang TCM Hospital Affiliated to Nanjing University of Chinese Medicine, Zhangjiagang, 215600 China; 3 Department of Endocrinology, Zhangjiagang TCM Hospital Affiliated to Nanjing University of Chinese Medicine, Zhangjiagang, 215600 China; 4 Department of Orthopedics, The First Affiliated Hospital of Soochow University, Suzhou, 215006 China; 5 Department of Gynecology, The First People’s Hospital of Zhangjiagang, Soochow University, Suzhou, 215006 China

**Keywords:** Glucocorticoid-induced bone loss, D1R, Osteoblast, ERK1/2, Osteoporosis

## Abstract

**Background:**

The inhibition of osteogenic differentiation is a major factor in glucocorticoid-induced bone loss, but there is currently no effective treatment. Dopamine, a major neurotransmitter, transmits signals via five different seven-transmembrane G protein-coupled receptors termed D1 to D5. Although the relevance of the neuroendocrine system in bone metabolism has emerged, the precise effects of dopamine receptor signaling on osteoblastogenesis remain unknown.

**Methods:**

In vitro, western blotting and immunofluorescence staining were used to observe the expression of dopamine receptors in MC3T3-E1 and BMSCs cells treated with dexamethasone (Dex). In addition, Alizarin red S (ARS) and alkaline phosphatase (ALP) staining and western blotting were used to evaluate the effect of D1R activation on osteogenic differentiation in Dex-induced MC3T3-E1 cells via the ERK1/2 signaling pathway. In vivo, micro-CT and hematoxylin and eosin (H&E), toluidine blue and immunohistochemical staining were used to determine the effect of D1R activation on Dex-induced bone loss.

**Results:**

We demonstrated that the trend in D1R but not D2-5R was consistent with that of osteogenic markers in the presence of Dex. We also demonstrated that the activation of D1R promoted Dex-induced osteogenic differentiation by activating the ERK1/2 pathway in vitro. We further demonstrated that a D1R agonist could reduce Dex-induced bone loss, while pretreatment with a D1R inhibitor blocked the effect of a D1R agonist in vivo.

**Conclusions:**

Activation of D1R promotes osteogenic differentiation and reduces Dex-induced bone loss by activating the ERK1/2 pathway. Hence, D1R could serve as a potential therapeutic target for glucocorticoid-induced osteoporosis.

**Supplementary Information:**

The online version contains supplementary material available at 10.1186/s10020-022-00453-0.

## Background

Glucocorticoids (GCs) have been widely used in the clinic, especially in alleviating inflammatory diseases. It has been indicated that the excessive use of GCs can lead to osteoporosis and osteonecrosis. GC-induced osteoporosis (GIOP) is a serious consequence leading to fractures, but there is currently no effective treatment (Tsourdi and Hofbauer 2018; Hardy et al. 2018). GCs can destroy the microstructure of bone and result in bone mass loss by inhibiting bone formation. The inhibition of the viability or differentiation of osteoblasts is one of the mechanisms of this bone loss. Therefore, the goal in treating GIOP is to reduce the inhibitory effect of GCs on osteoblasts.

Dopamine, a major neurotransmitter, transmits signals via five different seven-transmembrane G protein-coupled receptors termed D1 to D5, and these receptors fall into two groups. One group, the D1-like receptor family, consists of the D1 and D5 receptors, while the other group, the D2-like receptor family, consists of the D2, D3, and D4 receptors. Differential expression of the individual receptors at specific sites accounts for the different functional consequences of dopamine stimulation in the brain. Dopamine receptors are also expressed in the cardiovascular and renal systems. Iasevoli et al. (2013) showed that GC excess affects the mRNA expression and distribution of D1-like and D2-like receptors (albeit the latter to a lesser extent) and the neuropeptide enkephalin in cortico-striatal areas of the rat forebrain. Dopamine receptors are widely distributed in the central and peripheral nervous systems, have potential effects on nervous system diseases and are also therapeutic targets of various diseases. Recent studies have shown that dopamine receptors have many effects on the eyes, cardiovascular system, pancreas, tumor, etc. Furthermore, a few studies of dopamine receptor agonists and antagonists that modulate the functions of osteoblasts and osteoclasts have been reported (Nakashioya et al. 2011). Buckley et al. (2017) reported an inhibitory effect of chlorpromazine, a dopamine receptor antagonist, on bone formation via the suppression of osteoblastic cell functions in vivo and in vitro. Lee et al. (2015) showed that the mRNA and protein of all five dopamine receptors were expressed in MC3T3-E1 cells, and these functional dopamine receptors enhanced mineralization. Although previous studies reported that dopamine receptors were also expressed in osteoblasts, their function in this cell type, especially under GC-stimulated conditions, remains unclear.

**Scheme 1:**
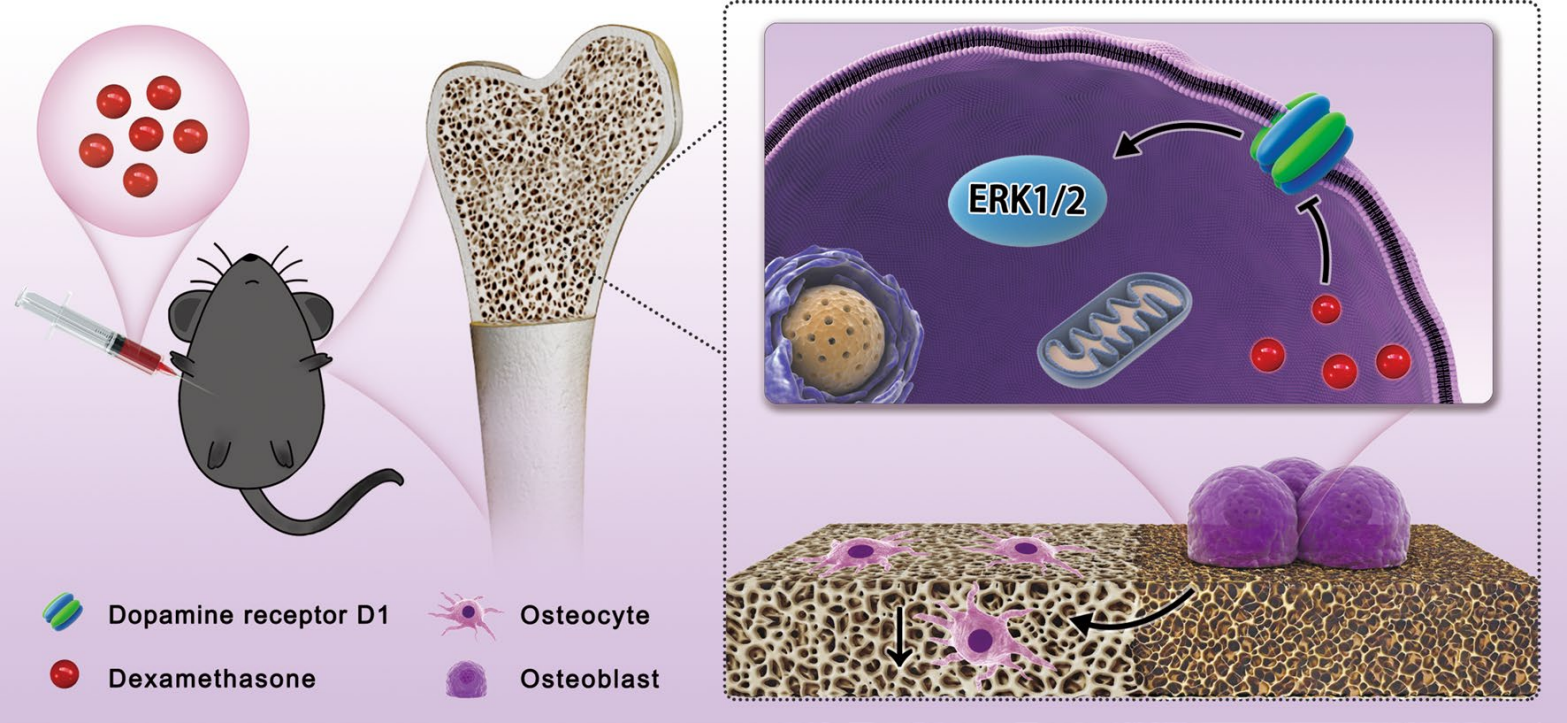
Dexamethasone inhibites osteogenic differentiation and induces bone loss by inhibiting the activation of dopamine receptor D1 via downregulating the ERK1/2 signaling pathway

In this study, we found that the expression of the D1 receptor (D1R) was inhibited by dexamethasone (Dex) dose-dependently in vivo and in vitro. In addition, our results demonstrated that a D1R agonist prevented Dex-induced osteoporosis by enhancing bone formation. Mechanistically, the D1R agonist protected osteoblasts from Dex by activating ERK1/2 signaling. Our results demonstrated the therapeutic potential of D1R in the treatment of GIOP (Scheme 1).

## Methods

### Cell culture and osteoblast differentiation

BMSCs cells and MC3T3-E1 cells (Cell Bank of the Chinese Academy of Sciences, Shanghai, China) were maintained in α-MEM with 10% fetal bovine serum in an incubator at 5% CO_2_ and 37 °C. Cells (1 × 10^6^ cells/well, 6-well plate) were incubated under osteogenic conditions (DMEM, 10% fetal bovine serum, 50 μg/ml ascorbic acid, 40 ng/ml Dex and 10 mM β-glycerophosphate) until reaching confluence and were then treated with or without 0.01–100 μM Dex (Sigma-Aldrich Inc., St Louis, MO, USA) for 12, 24 or 48 h or 3, 14 or 21 days, as indicated. The medium was changed every 3 days. To investigate whether D1R was responsible for the effect on Dex-mediated osteoblast differentiation, the first antagonist (0.2 nM SCH23390) was added for 10 min, then agonist (10 μM SKF38393) for 10 min before Dex in this experiment. To verified whether the ERK1/2 pathway was involved in the protective effect of activation of D1R to Dex-mediated osteoblast differentiation, the first antagonist (1 μM SCH772984) was added for 10 min, then agonist (10 μM SKF38393) for 10 min before Dex. The medium was changed once at 3 days, and the cells were allowed to differentiate for 14 or 21 days. After mineralized nodules had formed, ALP and ARS staining were performed.

### Cell viability assay

MC3T3-E1 cells (1 × 10^3^ cells/well) were plated in 96-well plates. The MC3T3-E1 were cultured in induction medium for 24 h and then treated with various concentrations of Dex, SKF38393, SCH23390 or SCH772984 for 1, 2, or 3 days. Ten microliters of CCK-8 (Beyotime, Shanghai, China) buffer was added to each well. After incubation at 37 °C and 5% CO_2_ for 1 h, absorbance was measured using a microplate reader (BioTek, Winooski, VT, USA) at a wavelength of 450 nm.

### ALP staining

BMSCs and MC3T3-E1 cells were incubated with an ALP stain. Briefly, the cells were washed three times with PBS. After fixation in 4% paraformaldehyde for 15 min, the cells were washed three times with PBS and then incubated in a BCIP/NBT working solution (Beyotime, Shanghai, China) in the dark for 30 min. The staining outcomes were observed under a microscope.

### ARS staining

To examine the matrix mineralization of osteoblasts, BMSCs and MC3T3-E1 cells were plated in 12-well plates at a concentration of 1 × 10^5^ cells per well in differentiation medium for 21 days. The cells were fixed with 4% paraformaldehyde at room temperature for 10 min. Then, the wells were rinsed three times with distilled water, and the cells were dyed with 1% ARS (Sigma, St Louis, MO, USA) at 37 °C for 30 min. Finally, the cells were washed thoroughly with distilled water three times. The staining outcomes were observed under a microscope.

### Immunofluorescence staining

MC3T3-E1 cells were cultured on 24-well plates, washed with PBS three times, fixed with 4% paraformaldehyde for 30 min and then permeabilized with Triton X-100 for 10 min. The cells were stained with primary antibodies against D1R (Abcam, ab20066, 1:1000) at 4 °C overnight, washed and incubated with secondary Alexa Fluor 555 antibodies (ab150078, Abcam 1:1000) and Molecular Probes Alexa Fluor 488 phalloidin (Cell Signaling Technology, Danvers, USA) for 1 h in the dark. After the cells were stained with DAPI for 10 min, the cells were imaged using a fluorescence microscope.

### Overexpression and silencing of D1R in MC3T3-E1 cells

Lentivirus packaging was made by OBiO Technology (Shanghai, China). Lentiviral shRNAs targeting D1R were obtained from Gene pharma and shRNA overexpression and silencing sequences are listed follow: pSLenti-CMV-Drd1-3FLAG-PGK-Puro (D1R): CMV-F: CGCAAATGGGCGGTAGGCGTG; MSCV-rev: CAGCGGGGCTGCTAAAGCGCATGC; pLKD-CMV-Puro-U6-shRNA (Drd1)_shDrd#1-TargetSeq: GGACTTTGTCTGTTCTCAT; pLKD-CMV-Puro-U6-shRNA(Drd1)_shDrd#2-TargetSeq: CCATTTCATCCTCCCTCAT; pLKD-CMV-Puro-U6-shRNA(Drd1)_shDrd#3-TargetSeq: CCACCACAGGTAATGGAAA. The MC3T3-E1cells were infected with the lentivirus at a MOI of 100 for 72 h following which stably transfected cell lines were obtained by culturing in medium containing puromycin according to the manufacturer's instructions.

### Western blot analysis

BMSCs and MC3T3-E1 cells were collected with 0.5% trypsin (GIBCO, Grand Island, NE, U.S.). The cells were lysed in radioimmunoprecipitation assay (RIPA; Beyotime) for 30 min at 4 °C, and then the supernatant was collected by centrifugation at 14,800 rpm at 4 °C. The supernatant was purified, and the total protein concentration was quantified using a bicinchoninic acid (BCA) protein kit (Beyotime, Shanghai, China). Twenty micrograms of total protein were separated using SDS-PAGE (New Cell & Molecular Biotech Co., Ltd., Suzhou, China) and then transferred to a PVDF membrane (Merck Millipore, Carrigtwohill, County Cork, Ireland). After being blocked with QuickBlock™ blocking buffer (Beyotime, Shanghai, China), the membranes were incubated with the appropriate primary antibodies overnight at 4 °C. Antibodies against runt-related transcription factor 2 (Runx2, Abcam, ab76956, 1:1000), osterix (OSX) (Abcam, ab22552, 1:1000), ALP (Abcam, ab95462, 1:1000), D1R (Invitrogen, PA5-27172, 1:1000), D2R (Abcam, ab191041, 0.1–0.5 µg/ml), D3R (Abcam, ab42114, 1:1000), D4R (Abclonal, A1337, 1:1000), D5R (Abclonal, A1719, 1:1000), total ERK1/2 (CST, 4695S, 1:1000), p-ERK1/2 (CST, 4377S, 1:1000), JNK (Abcam, ab179461, 1:1000), p-JNK (CST, 4668S, 1:1000), p38 (Abcam, ab170099, 1:1000), p-p38 (CST, 4511S, 1:1000), and β-actin (CST, 12262S, 1:1000) were used. The membranes were rinsed 3 times with TBS-Tween and then incubated at room temperature with secondary antibodies for 1 h. Enhanced chemiluminescence (Immobilon Western Chemiluminescent HRP Substrate; Millipore, Burlington, MA, USA) was used to visualize the protein bands, and the relative gray level was analyzed using ImageJ.

### Animal studies and drug applications

Eight-week-old C57BL/6 J mice were purchased from JOINN Laboratories (NO.2019110034, Suzhou, China). Animal experiments were approved by the Animal Ethics and Welfare Committee (AEWC) of Zhangjiagang TCM Hospital Affiliated to Nanjing University of Chinese Medicine (Approval date: 2019-10-10, Approval NO: AEWC -20191002). Briefly, the mice were randomly divided into five groups: (1) control (Ctrl) group, (2) vehicle group, (3) Dex + D1R agonist group, (4) Dex + D1R inhibitor + D1R agonist group and (5) Dex + D1R inhibitor group (n = 6 mice for each group). Mice in the vehicle group were injected with 20 mg/kg Dex. Mice in the Dex + D1R agonist group were administered 20 mg/kg Dex and 1 mg/kg SKF38393, and mice in the Dex + D1R inhibitor + D1R agonist group were administered 20 mg/kg Dex, 1 mg/kg SKF38393 and 1 mg/kg SCH23390. Mice in the Dex + D1R inhibitor group were administered 20 mg/kg Dex and 1 mg/kg SCH23390 (Mayerhofer et al. 1999). Mice in the Ctrl group were injected with an equal volume of normal saline. Drugs were administered by intraperitoneal injection every day for 4 weeks before sacrifice. Bilateral femurs, livers and kidneys were collected for subsequent analyses.

### Microcomputed tomography (micro-CT) analysis

The femurs (n = 6/groups) of the mice were measured by high-resolution micro-CT (SkyScan 1176, SkyScan, Knotich, Belgium). The femurs were scanned at 9 μm per layer, and the X-ray parameters were set at a voltage of 80 kV and a current of 100 mA. Bone mass density (BMD), bone volume per tissue volume (BV/TV), bone surface/bone volume (BS/BV), bone surface/total volume (BS/TV), trabecular number (Tb.N), trabecular thickness (Tb.Th) and connectivity density (Conn.Dn) were measured by the program CTAn (Bruker micro-CT, Kontich, Belgium). The relevant three-dimensional (3D) images were analyzed after processing.

### Histological and immunohistochemical analyses

After micro-CT analysis, the femurs were preserved at 4 °C for histological and immunohistochemical analyses. All femurs were decalcified with 15% ethylenediaminetetraacetic acid (EDTA, Sigma) for 4 weeks, embedded in paraffin blocks and sectioned at a thickness of 5 μm by a microtome. Hematoxylin and eosin (H&E), toluidine blue and immunohistochemical (IHC) staining were performed. Images were captured with an Olympus microscope.

### Statistical analysis

All experiments were performed at least 3 times. All values are expressed as the mean ± standard deviation (M ± SD). Fold changes for each treatment were compared with those of the respective control group at each designated time point. SPSS 25.0 software was used for statistical analysis in this study. One-way analysis of variance (ANOVA) was used for statistical comparisons among more than two groups. The post hoc Newman-Keuls test was suitable for comparisons of two groups. When the p value was less than 0.05, the difference was considered statistically significant.

## Results

### *Dex reduced D1R expression *in vitro

**Fig. 1:**
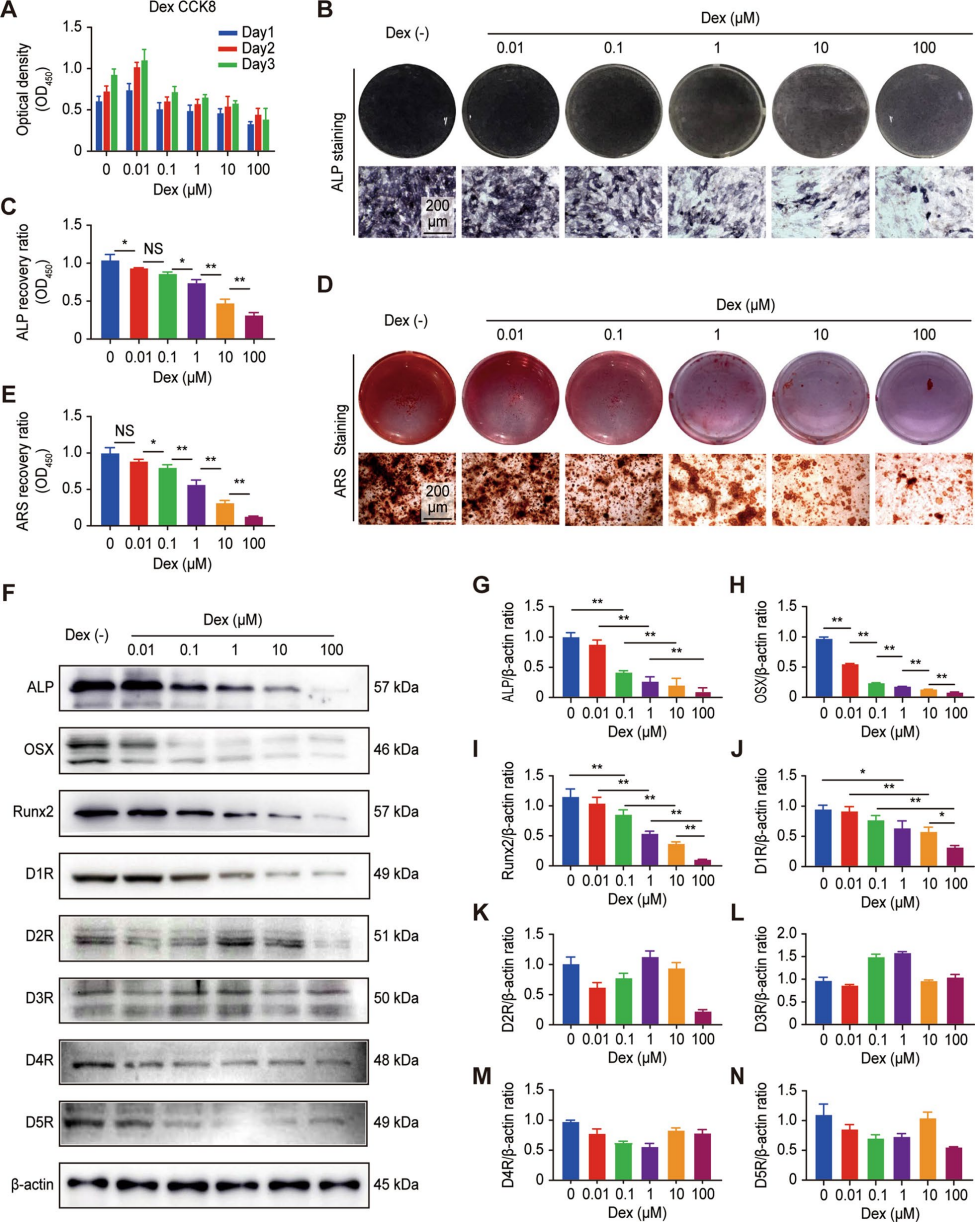
Dex inhibited the differentiation of osteoblasts and reduced D1R expression in vitro. **A** CCK-8 analysis of Dex-induced MC3T3-E1 cells. **B** Representative images showing ALP staining. Scale bar: 200 μm. **C** Quantitative analysis of ALP staining. n = 3 per group. NS: Not statistically significant, * p < 0.05, ** p < 0.01, vs. the Dex (−) group. **D** Representative images showing ARS staining. Scale bar: 200 μm. **E** Quantitative analysis of ARS staining. n = 3 per group. NS: Not statistically significant, * p < 0.05, ** p < 0.01, vs. the Dex (−) group. **F** Representative images of western blots probed with antibodies against the osteogenesis-specific proteins ALP, OSX, and Runx2 and the dopamine receptors D1–D5. **G**–**N** Quantification of the protein levels. n = 3 per group. NS: Not statistically significant, * p < 0.05, ** p < 0.01, vs. the Dex (−) group

To gain insight into Dex-induced osteogenesis inhibition, we examined the DR signaling pathway. To assess the effects of Dex on the proliferation of osteoblasts, the CCK-8 assay was used, and MC3T3-E1 cells were incubated with 0–100 μM Dex. The results indicated that exposure of MC3T3-E1 cells to Dex at concentrations ranging between 0 and 0.01 µM led to an increase in osteoblast proliferation, and when the concentration of Dex was greater than 0.01 μM, osteoblast proliferation was restrained in a dose-dependent manner (Fig. 1A). As evidenced by ALP and ARS staining, compared with those of the control group, the number of positive cells and calcium deposition decreased with increasing Dex concentrations (Fig. 1B and D). Quantitative analysis also indicated that ALP staining was more than 1 times lower at 100 μM Dex than at 0.1 μM Dex, while the trend in ARS staining was more obvious. When the Dex concentration was 100 μmol, the ARS staining was only 1/5 of that at 0.1 μM Dex (Fig. 1C and E). The results of ALP and ARS staining in BMSCs cells demonstrated the similar phenomena (Additional file 1: Figure S1A–C). We further studied the effect of Dex on the expression of the osteogenic-related proteins ALP, OSX and Runx2 in MC3T3-E1 cells by western blot analysis and found that Dex treatment significantly decreased the protein expression of ALP, OSX and Runx2 in a dose-dependent manner (Fig. 1F–I).

**Fig. 2:**
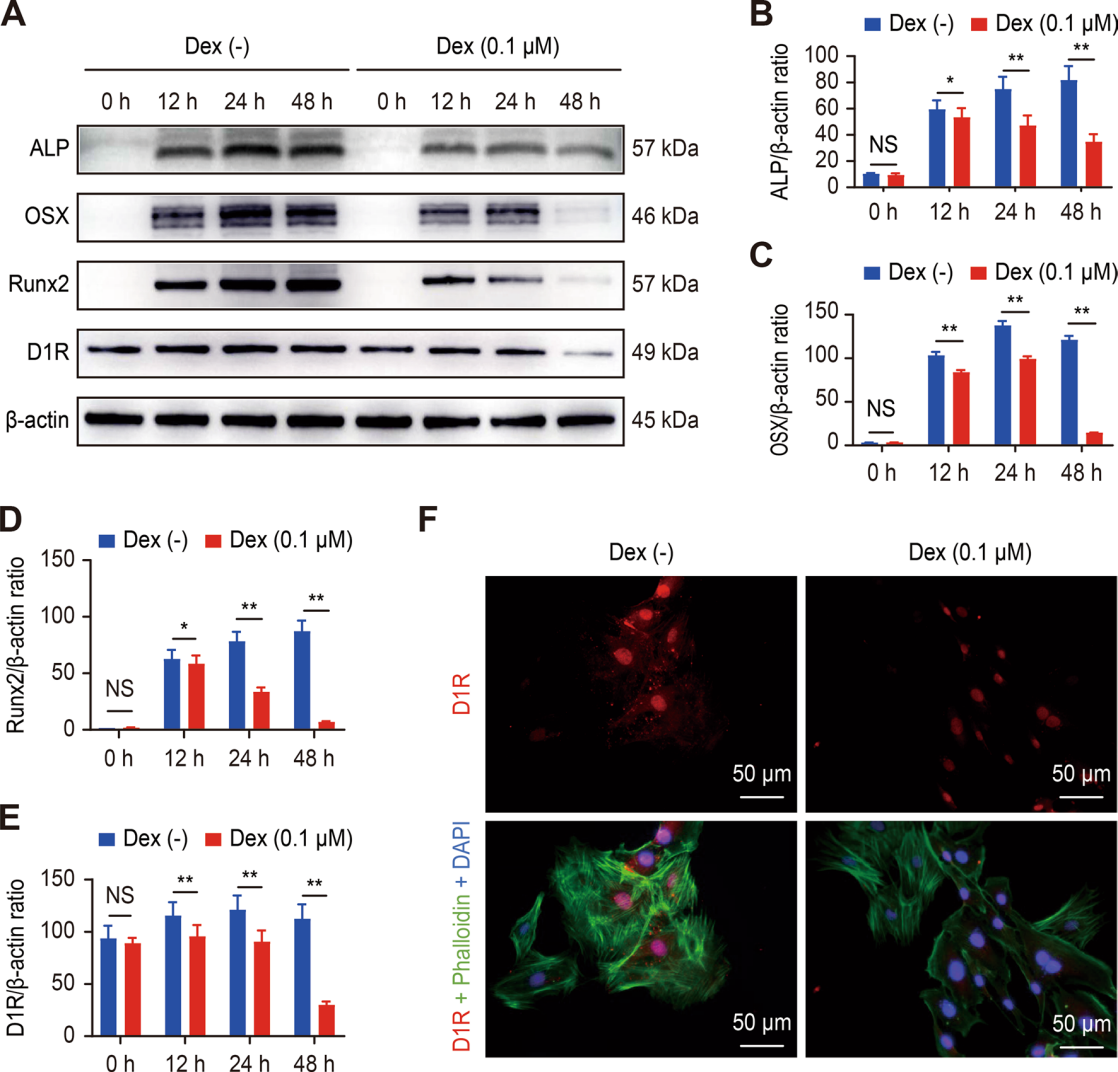
Dex inhibited the differentiation of osteoblasts and reduced D1R expression in vitro. **A** Representative images of western blots probed with antibodies against ALP, OSX, Runx2, and D1R. **B**–**E** Quantification of ALP, OSX, Runx2, and D1R protein levels. n = 3 per group. NS: Not statistically significant, * p < 0.05, ** p < 0.01, vs. the Dex (−) group at the same time point. **F** Representative images showing immunofluorescence staining of D1R. Scale bar: 50 μm

All dopamine receptor subtypes, D1R to D5R, were detectable by western blotting at similar levels (Fig. 1F and Additional file 1: Figure S1D). Interestingly, the trend in the expression of D1R was consistent with that of osteogenic-related protein markers in the presence of Dex (Fig. 1J and Additional file 1: Figure S1E), while the expression of D2-5R was not correlated with the concentration of Dex (Fig. 1K–N). In addition, as shown in Fig. 2A, we observed the expression of osteogenic-related proteins and D1R at different time points with or without Dex by western blot analysis. Over time, the expression of osteogenic-related proteins was more inhibited in response to Dex, while the trend in the change in D1R expression was consistent (Fig. 2B–E). Figure 2F shows that the fluorescence intensity of D1R was significantly decreased at 48 h after Dex administration. This finding suggests that Dex has a dose- and time-dependent effect on osteogenic differentiation and D1R expression.

### *Overexpression of D1R alleviated Dex-induced inhibition of osteoblast differentiation *in vitro

**Fig. 3:**
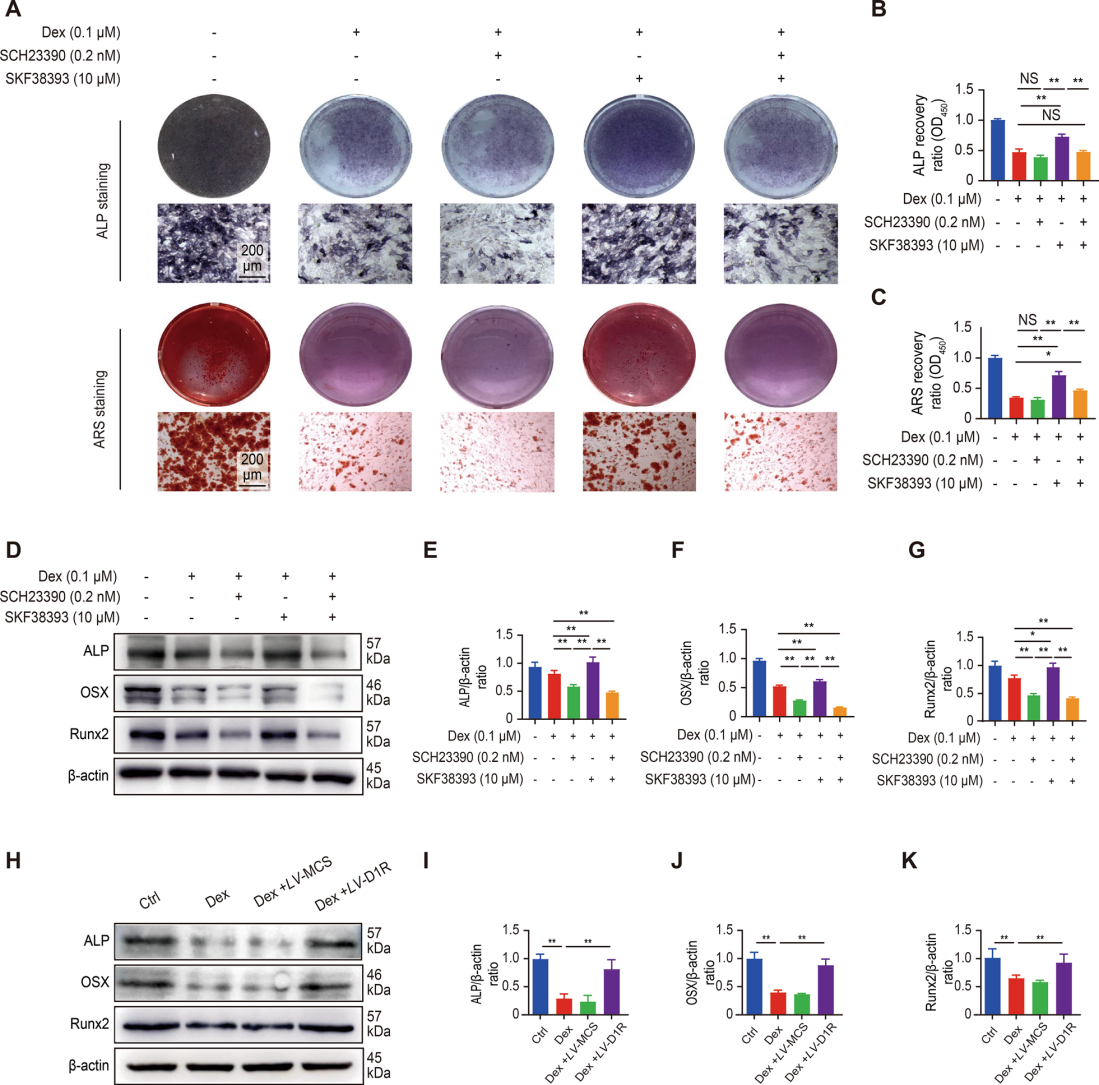
Activation of D1R alleviated Dex-induced inhibition of osteoblast differentiation in vitro. **A** Representative images showing ALP and ARS staining. Scale bar: 200 μm. **B** and **C** Quantitative analysis of ALP and ARS staining. n = 3 per group. NS: Not statistically significant, * p < 0.05, ** p < 0.01, vs. the control group. **D** Representative images of western blots probed with antibodies against ALP, OSX and Runx2. **E**–**G** Quantification of ALP, OSX and Runx2 protein levels. n = 3 per group. NS: Not statistically significant, * p < 0.05, ** p < 0.01, vs. the control group. **H** Representative images of western blots probed with antibodies against ALP, OSX and Runx2 in the overexpression experiment. **I**–**K** Quantification of ALP, OSX and Runx2 protein levels. n = 3 per group. NS: Not statistically significant, * p < 0.05, ** p < 0.01, vs. the control group

To investigate whether D1R was responsible for the effect on Dex-mediated osteoblast differentiation, SKF38393 (a specific D1R agonist) and SCH23390 (a specific D1R inhibitor) were used. As Additional file 1: Figure S2C to H shows, the activation of D1R protein expression was promoted by SKF38393 or D1R shRNA overexpression lentivirus, whereas SCH23390 or D1R shRNA silencing lentivirus had an inhibitory effect on D1R expression. We observed that compared Dex administration, overexpression of D1R attenuated the Dex-induced decrease in osteogenic differentiation. However, compared with agonist administration, the addition of SCH23390 (D1R inhibitor) weakened the protective effect of the agonist on Dex-induced osteogenesis inhibition, as evidenced by ALP and ARS staining (Fig. 3A–C and Additional file 1: Figure S3A–C). Furthermore, western blot analysis showed that the master osteogenic transcription factors ALP, OSX and Runx2 in osteoblasts were significantly downregulated by Dex. In contrast, the protein levels were reversed in the presence of SKF38393, but the effect was inhibited by the addition of SCH23390 (Fig. 3D–G and Additional file 1: Figure S3D–G). We also verified the results via overexpression or silencing the D1R by lentiviral transfection (Fig. 3H–K, Additional file 1: Figure S2I–L). Taken together, these data suggest that the activation of D1R alleviated Dex-induced inhibition of osteoblast differentiation in vitro.

### *The ERK1/2 pathway was involved in the protective effect of D1R against Dex-induced osteoblast activities *in vitro

**Fig. 4:**
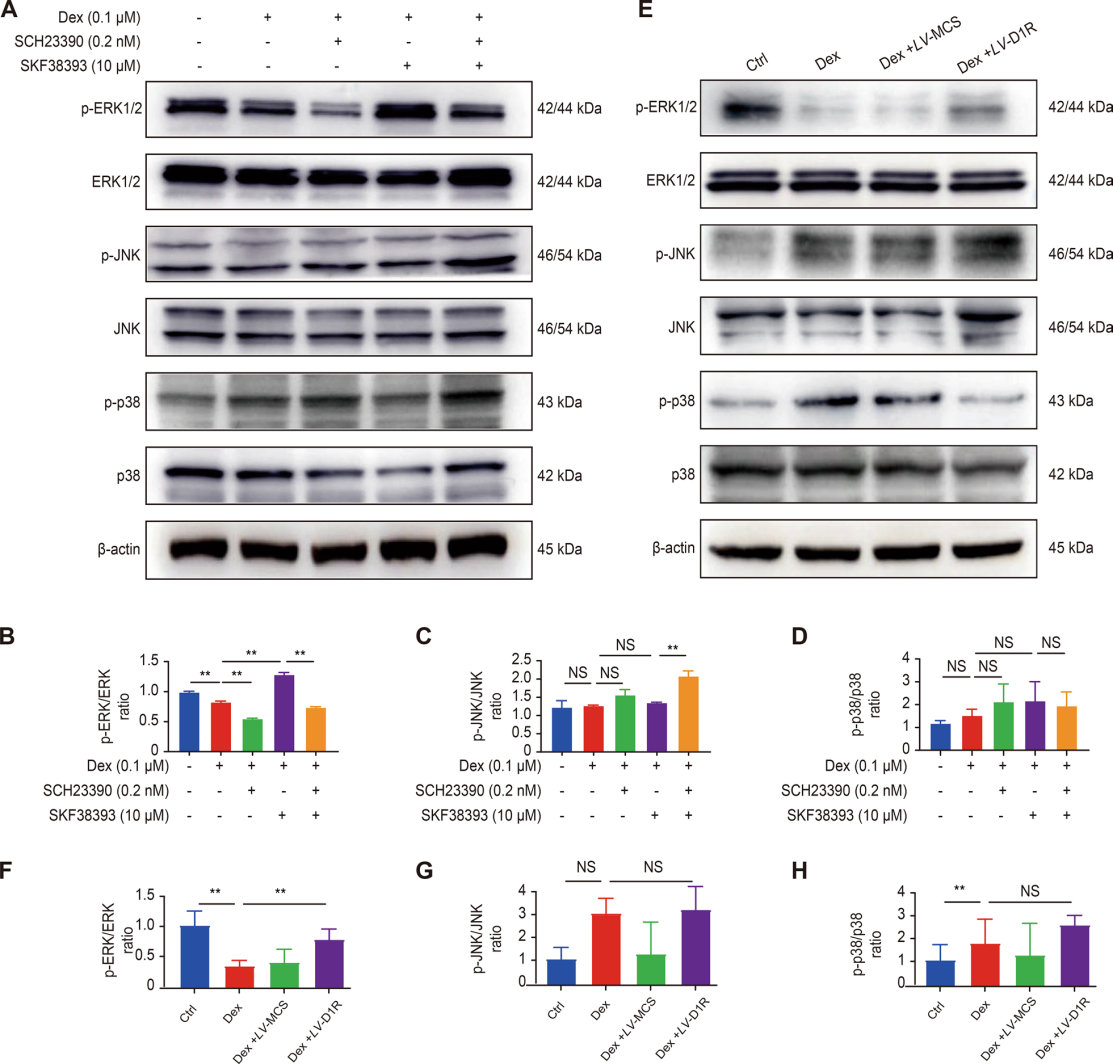
The ERK1/2 pathway was involved in the protective effect of D1R against Dex-induced osteoblasts in vitro. **A** Representative images of western blots probed with antibodies against the MAPK signaling pathway proteins p-ERK1/2, ERK1/2, p-JNK, JNK, p-p38, and p38 after 30 min’ Dex treatment. **B**–**D** The ratios of p-ERK1/2/ERK1/2, p-JNK/JNK, and p-p38/p38. n = 3 per group. NS: Not statistically significant, * p < 0.05, ** p < 0.01, vs. the control group. **E** Representative images of western blots probed with antibodies against the MAPK signaling pathway proteins p-ERK1/2, ERK1/2, p-JNK, JNK, p-p38, and p38 in the overexpression experiment. **F**–**H** The ratios of p-ERK1/2/ERK1/2, p-JNK/JNK, and p-p38/p38. n = 3 per group. NS: Not statistically significant, * p < 0.05, ** p < 0.01, vs. the control group

The mitogen-activated protein kinase (MAPK) pathway plays an important regulatory role in the pathogenesis of osteoporosis, and previous studies indicated that Dex inhibited osteoblast differentiation and proliferation by negatively regulating MAPK signaling (Caplan et al. 2017). Thus, we investigated whether the MAPK signaling pathway was involved in the effect of D1R on Dex-mediated osteoblast inhibition by western blot analysis. The results showed that of the MAPK family member ERK1/2 but not JNK or p38 was decreased by Dex stimulation. However, compared with that of the Dex group, pretreatment with the agonist or overexpression of D1R markedly alleviated the Dex-induced inhibitory effect on ERK phosphorylation, but this effect was abrogated by the addition of the D1R inhibitor (Fig. 4A–C and F). Moreover, the other two MAPK family members, JNK and P38, did not show any significant trends (Fig. 4D–G and H).

**Fig. 5:**
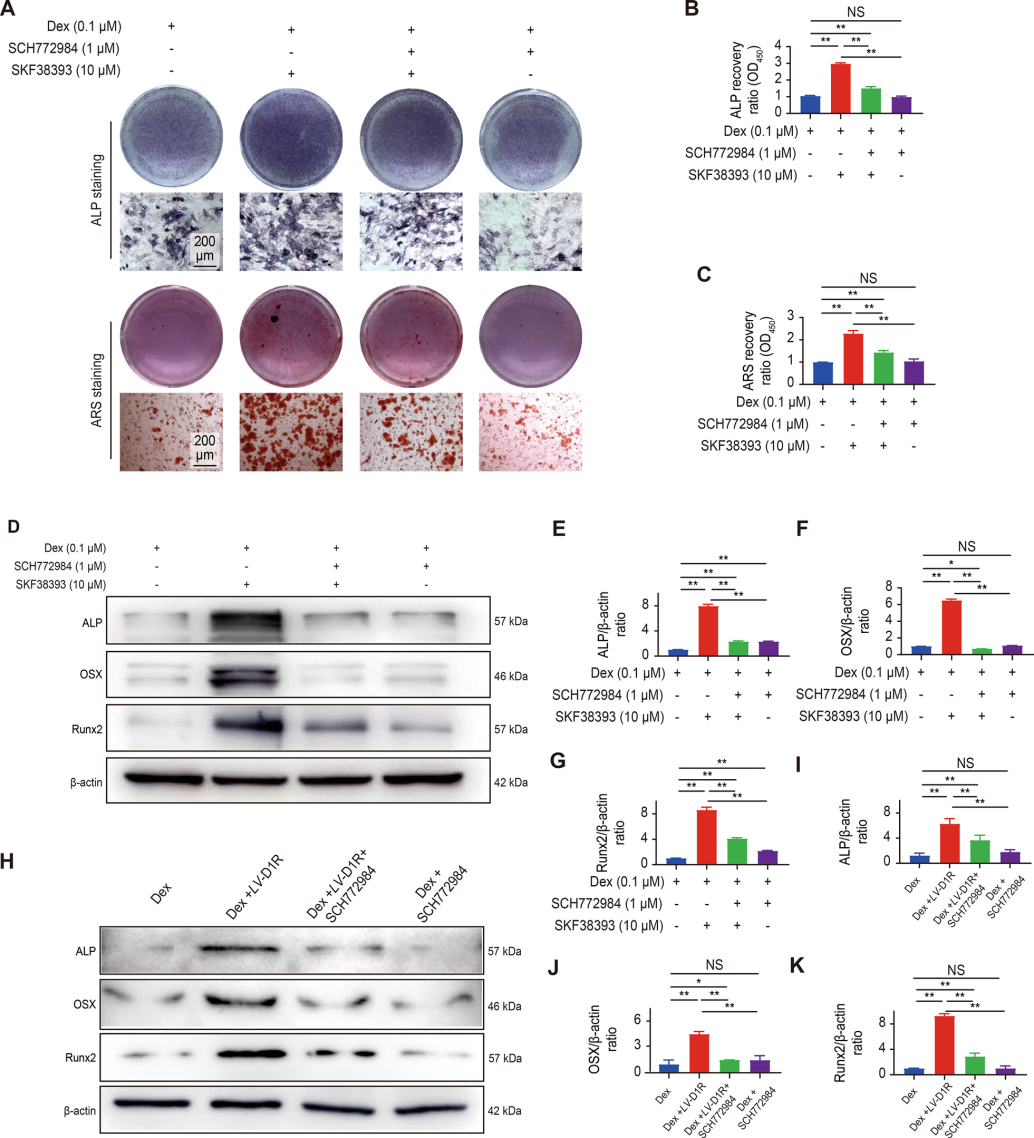
The ERK1/2 pathway was involved in the protective effect of D1R against Dex-mediated inhibition of osteoblast differentiation. **A** Representative images showing ALP and ARS staining. Scale bar: 200 μm. **B** and **C** Quantitative analysis of ALP and ARS staining. n = 3 per group. NS: Not statistically significant, * p < 0.05, ** p < 0.01, vs. the control group. **D** Representative images of western blots probed with antibodies against ALP, OSX and Runx2. **E**–**G** Quantification of ALP, OSX and Runx2 protein levels. n = 3 per group. NS: Not statistically significant, * p < 0.05, ** p < 0.01, vs. the control group. **H** Representative images of western blots probed with antibodies against ALP, OSX and Runx2. **I**–**K** Quantification of ALP, OSX and Runx2 protein levels in the overexpression experiment. n = 3 per group. NS: Not statistically significant, * p < 0.05, ** p < 0.01, vs. the control group

Next, we further verified that the ERK1/2 pathway was involved in the protective effect of activation of D1R to Dex-mediated osteoblast differentiation. After the pretreatment of MC3T3-E1 cells with a novel, specific ERK1/2 inhibitor (SCH772984 1 μM), western blot analysis showed that the expression of ERK1/2 in osteoblasts was decreased, and the proliferation of osteoblasts was not inhibited (Additional file 1: Figure S4A–C). The ALP staining results showed that pretreatment with the D1R agonist significantly increased the area of blue precipitate in the cytoplasm, while pretreatment with the specific ERK1/2 inhibitor reduced the area of blue precipitate in the cytoplasm. ARS staining revealed that compared with that of the D1R agonist group, less calcium deposition was observed (Fig. 4A and Additional file 1: Figure S4D) pretreated with the ERK1/2 inhibitor. Quantitative analysis also indicated that the D1R agonist group exhibited increased osteogenesis (twofold vs the ERK1/2 inhibitor group), and as we expected, similar ARS staining results were also obtained (Fig. 4B and C, Additional file 1: Figure S4E and F). Furthermore, the western blot results showed that when ERK1/2 inhibitors were added in combination with the D1R agonist or D1R shRNA overexpression lentiviral transfection, the inhibitor blocked the protection of the overexpression or agonist of D1R on Dex-induced osteogenesis inhibition in MC3T3-E1 cells (Fig. 5D–K). The specific JNK and P38 inhibitor (JNK-IN-8 and SB203580) were used to assess the effects of D1R agonist on the alleviating Dex-induced inhibition of osteoblast differentiation in vitro (Additional file 1: Figures S5 and S6). Taken together, these results suggested that the ERK1/2 pathway was involved in the protective effect of activation of D1R to Dex-mediated osteoblast differentiation and the JNK and p38 pathways may have no synergistic effects.

### *Activation of D1R promoted bone formation and alleviated Dex-induced osteoporosis *in vivo

**Fig. 6:**
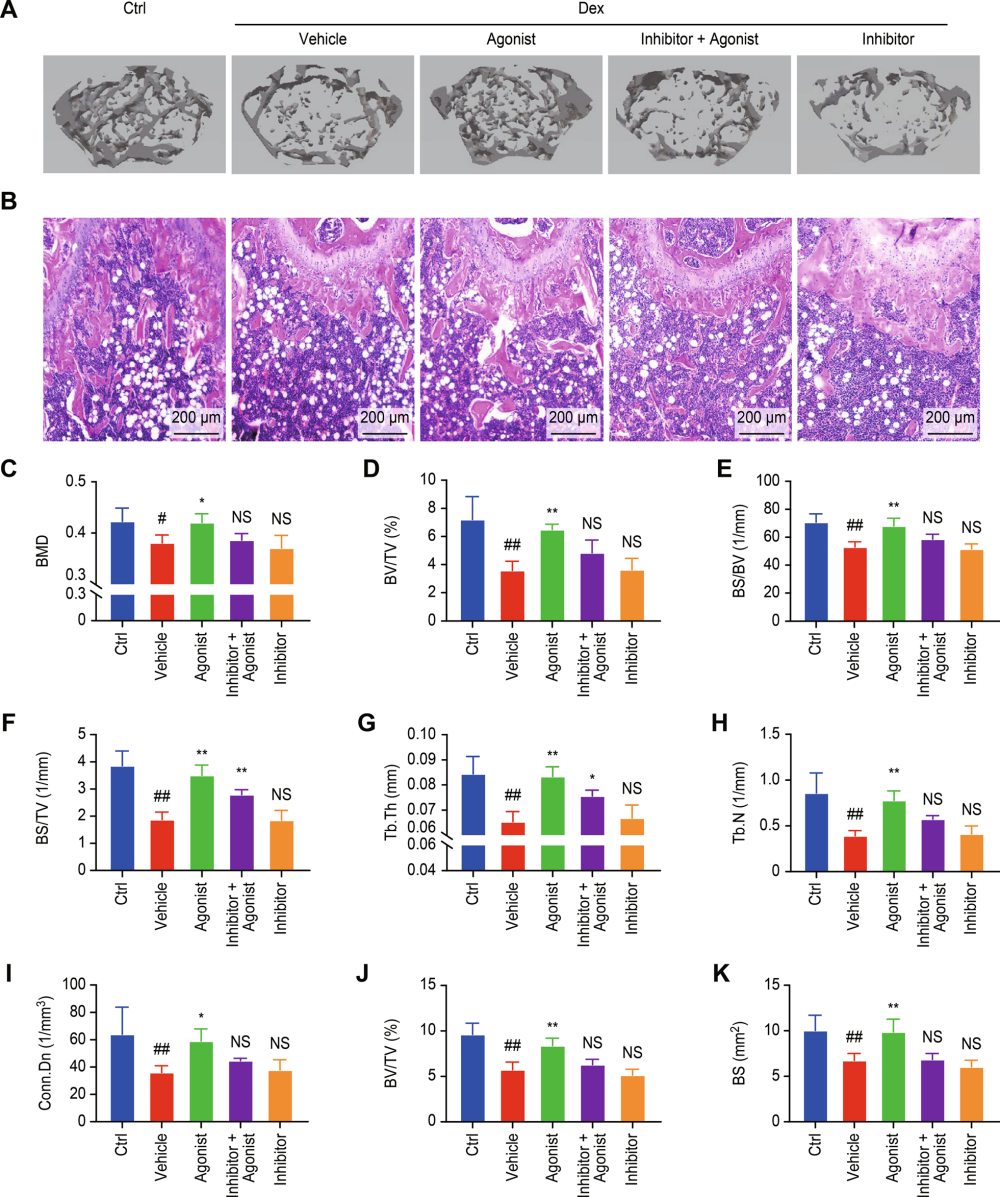
Activation of D1R alleviated Dex-induced osteoporosis in vivo. **A** Representative 3D reconstructions of μCT images and **B** representative paraffinized sections stained with H&E. **C** BMD within the region of interest (ROI) was calculated by μCT. **D** BV/TV **E** BS/BV, **F** BS/TV, **G** Tb. Th, **H** Tb. N. and **I** Conn.Dn. **J** and **K** BV/TV and BS within the ROI were analyzed by H&E staining. n = 5 per group. NS: Not statistically significant, * p < 0.05, ** p < 0.01, vs. the vehicle group, ^#^ p < 0.05, ^##^ p < 0.01, vs. the control group

Next, we further investigated the effects of D1R on Dex-induced osteoporosis in vivo. Representative μCT images of trabecular microstructures in the five groups of mice showed that Dex treatment induced an obvious loss of bone mass compared with that of mice in the control groups, but bone mass in mice in the agonist group was increased. In addition, pretreatment with the D1R inhibitor blocked the effects of the D1R agonist (Fig. 6A). Quantitative analysis demonstrated that activation of D1R significantly reduced Dex-induced bone loss, as shown by the following data (Ctrl group vs vehicle group vs agonist group vs agonist + inhibitor group): BMD (0.423 ± 0.026 vs 0.381 ± 0.015 vs 0.421 ± 0.017 vs 0.386 ± 0.012, respectively, g/cm^3^), BV/TV (7.206 ± 1.631 vs 3.588 ± 0.652 vs 6.489 ± 0.381 vs 4.842 ± 0.904, respectively, %), BS/BV (70.755 ± 5.919 vs 52.928 ± 3.802 vs 68.018 ± 5.510 vs 58.665 ± 3.486, respectively, 1/mm), BS/TV (3.855 ± 0.540 vs 1.872 ± 0.276 vs 3.503 ± 0.375 vs 2.794 ± 0.180, respectively, 1/mm), Tb.Th (0.084 ± 0.007 vs 0.065 ± 0.004 vs 0.083 ± 0.004 vs 0.076 ± 0.002, respectively, mm), Tb.N (0.859 ± 0.219 vs 0.392 ± 0.055 vs 0.779 ± 0.103 vs 0.573 ± 0.038, respectively, 1/mm), and Conn.Dn (63.946 ± 19.808 vs 36.006 ± 4.919 vs 59.005 ± 8.833 vs 44.652 ± 1.748, respectively, 1/mm^2^) (Fig. 6C–I).

Consistent with the μCT results, H&E staining of the femur tissue indicated that the D1R agonist alleviated Dex-induced bone mass loss (Fig. 6B). Quantification of the H&E staining results showed that compared with those of the ctrl group, the BV/TV and BS in the vehicle group were significantly reduced, but the BV/TV and BS were increased in the D1R agonist group (Fig. 6J and K). These results showed that activation of D1R prevented Dex-induced bone mass loss.

**Fig. 7:**
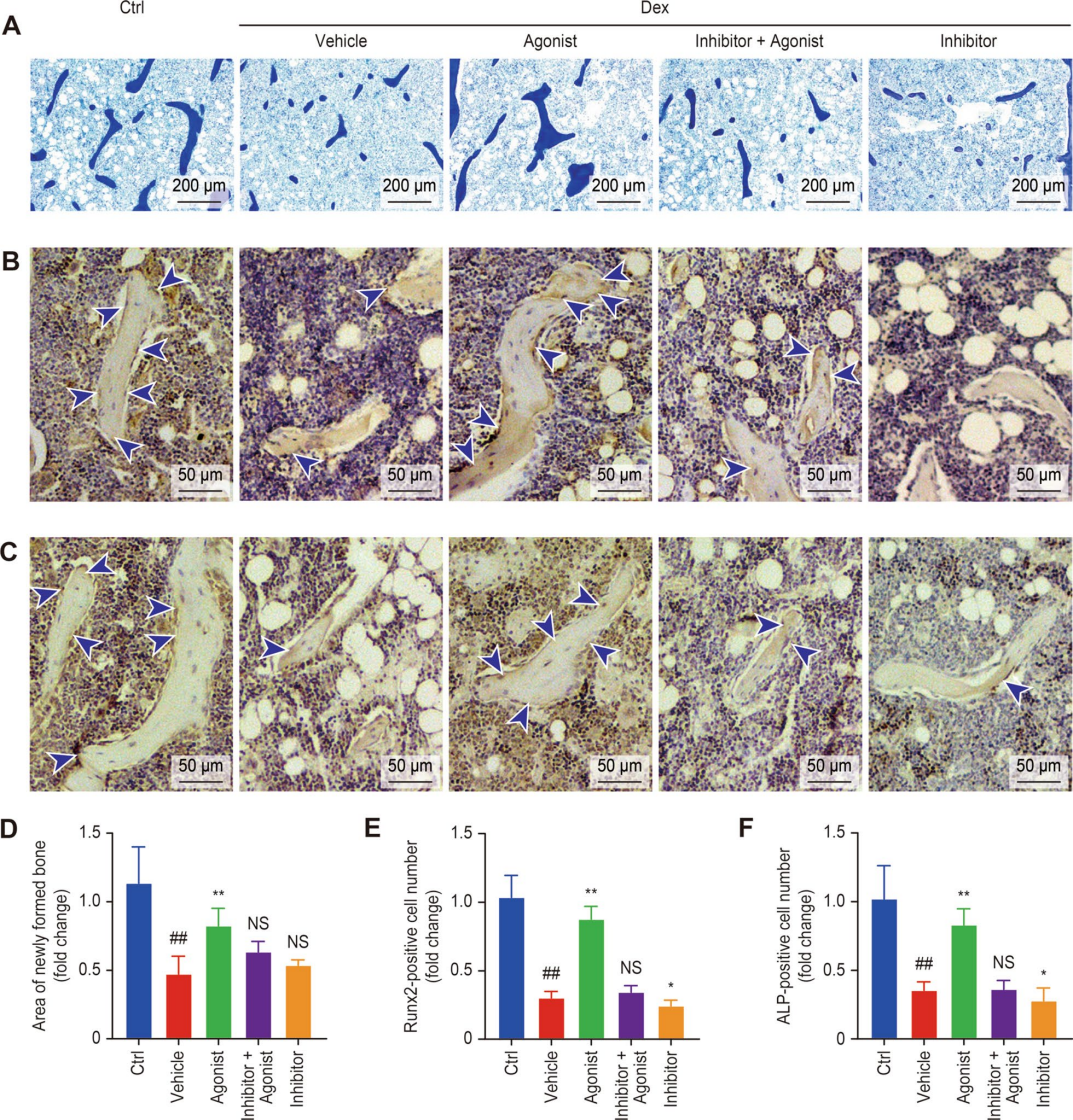
Activation of D1R promoted bone formation in vivo. **A** Representative images of toluidine blue staining. **B** and **C** Representative images showing IHC staining of Runx2 and ALP. **D** Quantitative analysis of the area of newly formed bone calculated by toluidine blue staining. **E** and **F** Quantitative analysis of the number of positive cells stained with Runx2 and ALP were analyzed by IHC staining, n = 5 per group. NS: Not statistically significant, * p < 0.05, ** p < 0.01, vs. the vehicle group, ^#^ p < 0.05, ^##^ p < 0.01, vs. the control group

Toluidine blue staining was used to determine whether the D1R agonist attenuated Dex-induced bone loss through the activation of bone formation. The results showed that Dex inhibited the rate of bone formation, and the addition of the D1R agonist enhanced bone formation. This effect was significantly inhibited with the addition of the D1R inhibitor (Fig. 7A and D). In addition, we assessed the osteogenic markers ALP and Runx2 by immunohistochemistry (Fig. 7B and C). As shown in Fig. 7E and F, the relative number of positive cells was decreased in the Dex-induced group compared with the control group and was increased in the agonist group. Finally, with the addition of the inhibitors, the number of IHC-positive cells was reduced. Interestingly, the number of positive cells in the inhibitor group was reduced compared to that in the vehicle group. The IHC staining results further support that D1R activation attenuates Dex-mediated inhibition of bone formation.

In conclusion, these results suggest that D1R activation could promote bone formation and protect against Dex-induced bone loss.

## Discussion

Long-term GCs have been widely used in clinical practice although a large number of studies have shown that it may be the main cause of bone loss (Ronchetti et al. 2018; Vandewalle et al. 2018; Kim et al. 2015), the specific pathogenesis is still unclear, and there is still no effective treatment. Previous studies have shown that the clinical use of GCs can reduce bone formation indicators, including osteocalcin, ALP and the carboxy-terminal peptide of type I collagen, while bone absorption indicators remain unchanged or are slightly decreases (Yang et al. 2020). It was found that bone density decreased in mice treated with prednisolone, and the proliferation and differentiation of osteoblasts were inhibited, leading to a decrease in the number of mature osteoblasts with stromal-secreting functions and osteoblasts that support osteoclasts (Han et al. 2019).

Osteoblasts have specific receptors and high affinity for Dex, and a hyperphysiological dose of GCs inhibits the proliferation, differentiation and function of osteoblasts (Rauch et al. 2010; Chotiyarnwong and McCloskey 2020; Adami and Saag 2019), which is the main cause of Dex-induced bone tissue damage (Balasubramanian et al. 2016). In recent years, studies showed that GCs may inhibit osteoblast differentiation and bone formation through the expression of the key transcription factor Cbfa1 (Hojo and Ohba 2020), the inhibition of the Wnt/β-catenin pathway (Belaya et al. 2018; Yin et al. 2015), the inhibition of IGF, and through osteoblast-associated factors such as BMP mRNA expression (Alkharobi et al. 2016; Morimoto et al. 2017). Our study confirmed that Dex could reduce bone density and new bone formation in mice, further indicating that Dex may cause bone loss by inhibiting osteogenesis. Interestingly, in the study we also showed that Dex reduced the expression of D1R, while the activation of D1R could weaken Dex-mediated inhibition of bone formation. In vivo studies have shown that the activation of D1R can promote the formation of new bone and alleviate Dex-induced bone destruction. These results suggest that D1R may play an important role in Dex-induced reductions in osteogenic function.

Dopamine is an important catecholamine neurotransmitter that is widely found in peripheral tissues and the central nervous system (Berke 2018). Through it is corresponding receptor, dopamine plays an important role in the physiological and pathological processes of various tissues. Studies have shown that the differentiation of osteoblasts was regulated by the nervous system. Additionally studies have confirmed that the differentiation of osteoblasts involving neurotransmitters plays a role in the development and repair of bones (Idelevich and Baron 2018). Lee et al. (2015) found that dopamine receptors (D1R-D5R) were expressed in osteoblasts and direct intervention can promote the proliferation and mineralization of osteoblasts. In this study, we also found D1R–D5R were expressed in MC3T3-E1 osteoblasts which was consistent with previous reports. However, we further found that the expression of D1R, ALP, Runx2, and OSX in osteoblasts was decreased with Dex administration, but dopamine receptors other than D1R were not affected. This difference may be because we treated MC3T3-E1 cells with Dex. Furthermore, we found that activation of D1R receptor could relieve decrease in differentiation of osteoblasts induced by DEX. Interestingly, it was consistent with Wang et al. (2020)’ report which dominant role of the D1 receptor on BMSC osteogenic differentiation. These results suggest that D1R but not D2R may be related to the Dex-induced decrease in osteogenic function.

MAPKs belong to the serine-threonine protein kinase family, mainly include the ERK1/2, JNK, and P38 pathways, and can guide cellular responses to various stimuli, regulating a variety of cellular functions, including proliferation, differentiation. Xie et al. (2019) found that Geniposide can alleviate GC-induced osteogenic inhibition of MC3T3-E1 cells by activating ERK signaling pathway through GLP-1 receptor. Our results also showed that the phosphorylation of ERK1/2 in MC3T3-E1 cells decreased in response to Dex, which inhibited the differentiation and proliferation of osteoblasts. Ding et al. (2015) showed that Dex can induce JNK phosphorylation mediated by activation of osteoblasts and bone cells and promote apoptosis, Yu et al. (2016) founded that neuropeptide Y1 receptor signaling pathway mediates the harmful effects of glucocorticoid on osteoblast differentiation by inhibiting ERK signaling pathway, but the expression of p38 signal is not affected in this process. In our experiment, Dex was not found to play a role in the p38 MAPK and JNK pathways in osteoblasts. This may be related to the sensitivity of different cell lines to Dex under different culture conditions, which may affect pathways based on sex differences, and the specific mechanism needs further research.

However, this study has the following limitations. Bone metabolism includes two major processes: osteoblast-mediated bone formation and osteoclast-mediated bone absorption, which are not independent and are regulated by the interaction and influence of osteoblasts and osteoclasts (Ping et al. 2017). Our study focused on the effect of changes in dopamine receptors on osteoblasts in the context of GC-induced bone loss. Although some studies have confirmed the influence of DRs on osteoclasts (Hanami et al. 2013; Yang et al. 2016; Kentaro et al. 2013), it is not clear whether DRs influence osteoclasts in the context of GC-induced bone loss. Our team is conducting follow-up studies on related aspects. In this study, rodents such as mice may be far behind humans. In subsequent studies, we will continue to conduct in-depth analyses on the above issues and further clarify the role of D1R in Dex-mediated regulation of bone remodeling by studying large animals or primates in vivo.

In summary, we found that after Dex administration, mice lost bone mass, bone density decreased, osteoblast differentiation and function were affected, and osteoblast D1R expression was inhibited. Studies have shown that Dex inhibits the expression of D1R but not D2-5R in osteoblasts by reducing the phosphorylation level of the ERK1/2 pathway, thus inhibiting the differentiation of osteoblasts and affecting the function of osteoblasts. D1R agonists can activate the ERK1/2 pathway and significantly reduce the inhibitory effect of Dex on osteoblasts, while ERK1/2 pathway inhibitors can inhibit the protective effect of D1R agonists on osteoblasts. Thus, understanding the direct regulation of D1R on osteoblasts and its mechanism will help to provide a new therapeutic strategy for GC-induced bone loss and provide new ideas to better understand the relationship between neuropsychiatric diseases and osteoporosis.

## Supplementary Information

**Additional file 1: Figure supplements**.** Figure S1**. Dex inhibited the differentiation of osteoblasts and reduced D1R expression in Rat BMSCs cells.** Figure S2**. Modulates the expression of D1R in MC3T3-E1 cells.** Figure S3**. Activation of D1R alleviated Dex-induced inhibition of osteoblast differentiation in Rat BMSCs cells.** Figure S4**. ERK1/2 mediated the protective effect of D1R against Dex-mediated inhibition of osteoblast differentiation in Rat BMSCs cells.** Figure S5**. The JNK pathway has no synergistic effects with ERK1/2 in the protective effect of activation of D1R to Dex-mediated osteoblast differentiation.** Figure S6**. The p38 pathway has no synergistic effects with ERK1/2 in the protective effect of activation of D1R to Dex-mediated osteoblast differentiation.** Figure S7**. H&E staining of the liver and kidney after treatment in vivo.

## Data Availability

The date used to support the findings of this study are available from the corresponding author upon request.

## Abbreviations

GCs: Glucocorticoids
GIOP: GC-induced osteoporosis
D1R: Dopamine 1 receptor
Dex: Dexamethasone
ALP: Alkaline phosphatase
ARS: Alizarin red S
RIPA: Radioimmunoprecipitation assay
Runx2: Runt-related transcription factor 2
EDTA: Ethylenediaminetetraacetic acid
H&E: Hematoxylin and eosin
IHC: Immunohistochemical
MAPK: Mitogen-activated protein kinase
BV/TV: Bone volume per tissue volume
BS/BV: Bone surface/bone volume
BS/TV: Bone surface/total volume
Tb.N: Trabecular number
Tb.Th: Trabecular thickness
OGD/R: Oxygen–glucose deprivation/reoxygenation
