# Supplementary material for: Activation of dopamine receptor D1 promotes osteogenic differentiation and reduces glucocorticoid-induced bone loss by upregulating the ERK1/2 signaling pathway
Source: Mol Med. 2022 Feb 21;28:23. doi: 10.1186/s10020-022-00453-0 (PMC8862482; doi:10.1186/s10020-022-00453-0)
Supplement: Supplementary file 1 — Additional file 1: Figure supplements. Figure S1. Dex inhibited the differentiation of osteoblasts and reduced D1R expression in Rat BMSCs cells. Figure S2. Modulates the expression of D1R in MC3T3-E1 cells. Figure S3. Activation of D1R alleviated Dex-induced inhibition of osteoblast differentiation in Rat BMSCs cells. Figure S4. ERK1/2 mediated the protective effect of D1R against Dex-mediated inhibition of osteoblast differentiation in Rat BMSCs cells. Figure S5. The JNK pathway has no synergistic effects with ERK1/2 in the protective effect of activation of D1R to Dex-mediated osteoblast differentiation. Figure S6. The p38 pathway has no synergistic effects with ERK1/2 in the protective effect of activation of D1R to Dex-mediated osteoblast differentiation. Figure S7. H&E staining of the liver and kidney after treatment in vivo. [file 10020_2022_453_MOESM1_ESM.docx]

**Activation of dopamine receptor D1 promotes osteogenic differentiation and reduces glucocorticoid-induced bone loss by upregulating the ERK1/2 signaling pathway**

**Author names**

*Jie Zhu1,2***; Chengcheng Feng2,3***; Weicheng Zhang4***; Mengdan Zhong2,3; Wenkai Tang1,2; Zhifang Wang1; Haiwei Shi1; Zhengyu Yin1; Jiandong Shi1; Yu Huang5; Long Xiao1,2,4#; Dechun Geng4#; Zhirong Wang1,2#*

**Affiliations**

1.Department of Orthopedics, Zhangjiagang TCM Hospital Affiliated to Nanjing University of Chinese Medicine, Zhangjiagang, 215600, China;

2. Center Laboratory, Zhangjiagang TCM Hospital Affiliated to Nanjing University of Chinese Medicine, Zhangjiagang, 215600, China;

3. Department of Endocrinology, Zhangjiagang TCM Hospital Affiliated to Nanjing University of Chinese Medicine, Zhangjiagang, 215600, China;

4. Department of Orthopedics, The First Affiliated Hospital of Soochow University, Suzhou, 215006, China;

5. Department of Gynecology, Department of Gynecology, The First People's Hospital of Zhangjiagang, Soochow University, Suzhou, 215006, China.

*First authors with equal contribution.

**Corresponding author**

Long Xiao, Email: [zjgfy_spine_xl@njucm.edu.cn](mailto:zjgfy_spine_xl@njucm.edu.cn); Dechun Geng, Email: [szgengdc@suda.edu.cn](mailto:szgengdc@suda.edu.cn); Zhirong Wang, Email: zjgfy_spine_wzr@njucm.edu.cn.

**Supplementary material**

**
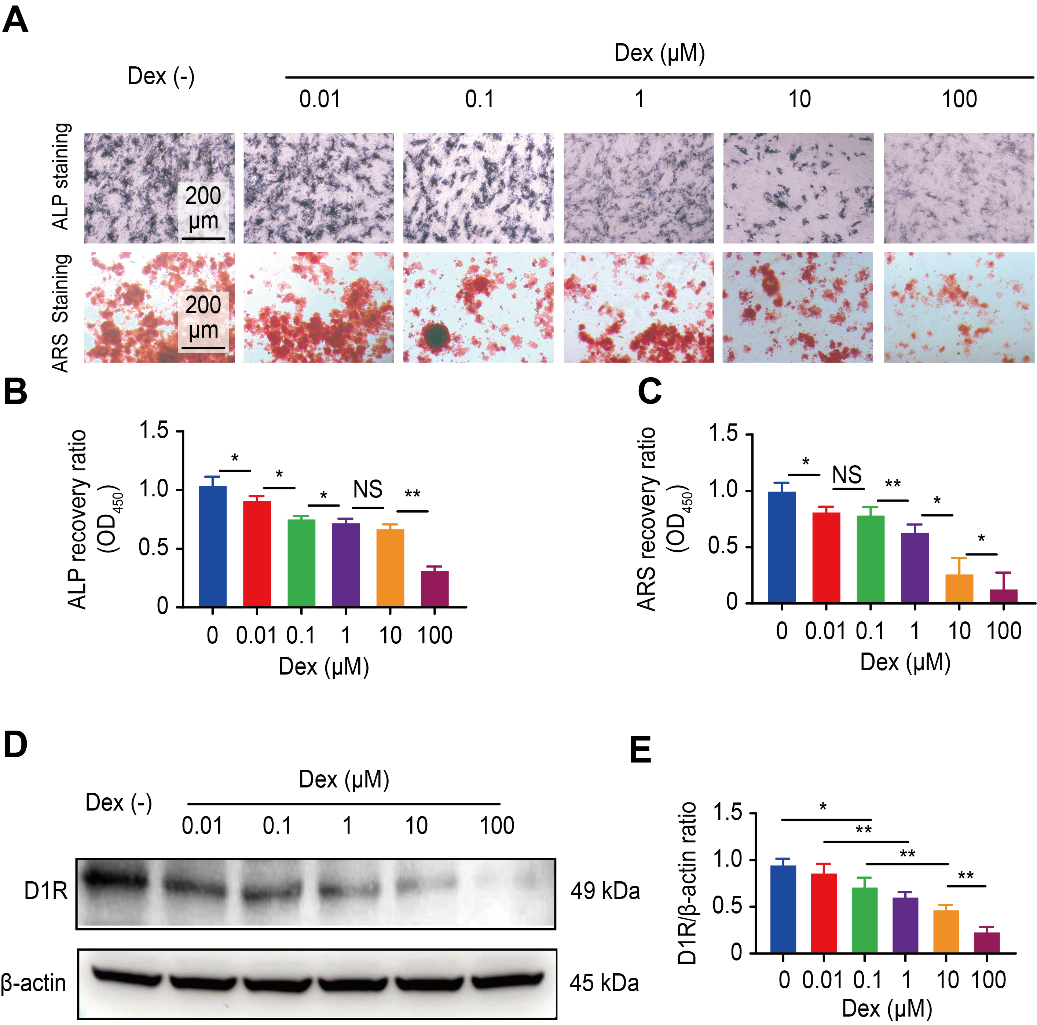
**

**Figure S1. Dex inhibited the differentiation of osteoblasts and reduced D1R expression in Rat BMSCs cells.** A) Representative images showing ALP and ARS staining. Scale bar: 200 μm. B and C) Quantitative analysis of ALP and ARS staining. n=3 per group. NS: Not statistically significant, * p <0.05, ** p <0.01, vs. the Dex (-) group. D) Representative images of western blots probed with antibodies against the dopamine receptors D1. E) Quantification of the protein levels. n=3 per group. NS: Not statistically significant, * p <0.05, ** p <0.01, vs. the Dex (-) group.

**
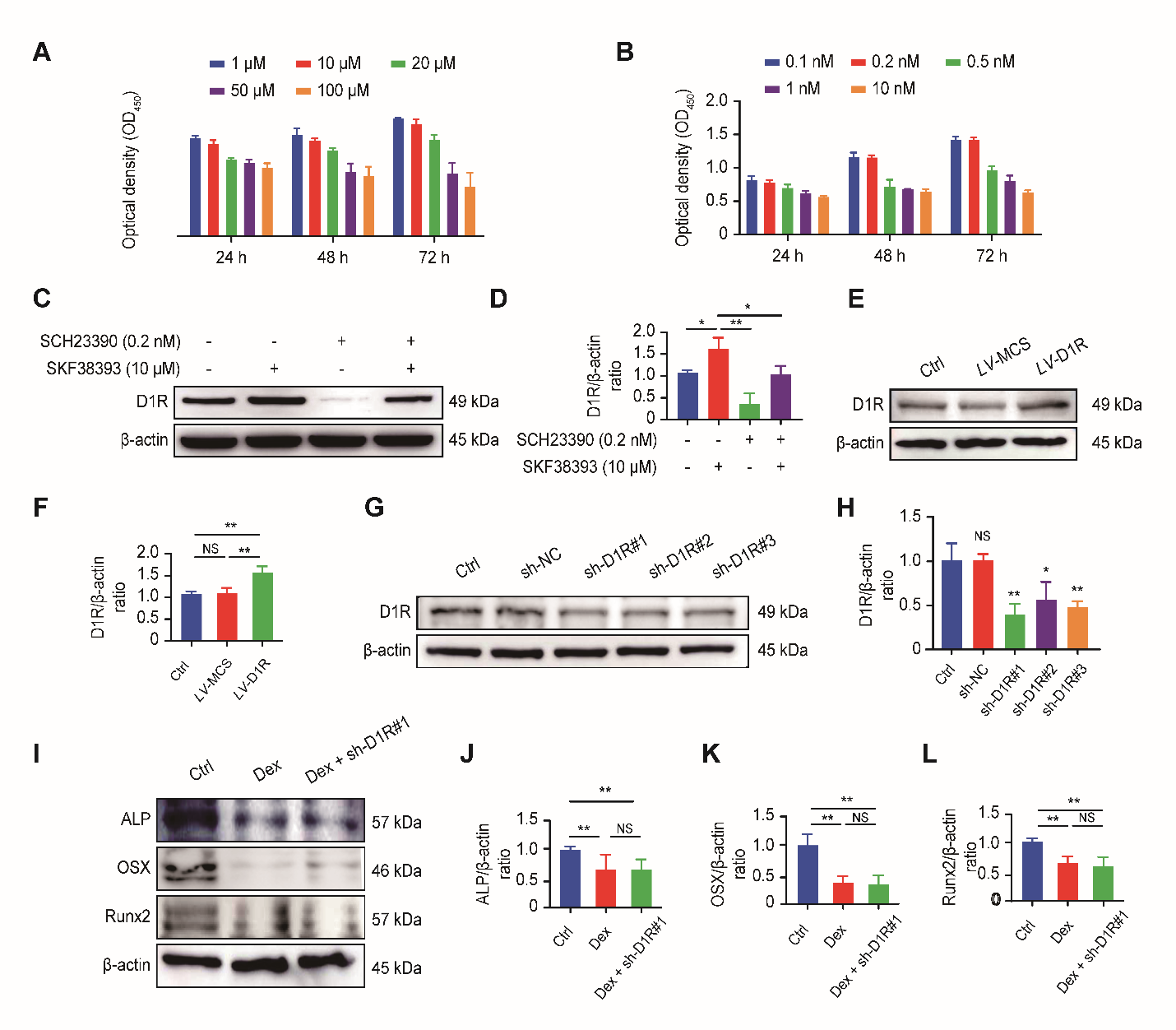
**

**Figure S2. Modulates the expression of D1R in MC3T3-E1 cells.** A and B) CCK-8 analysis of MC3T3-E1 cells treated with SKF38393 and SCH23390. C) Representative images of western blots probed with antibodies against D1R. D) Quantification of D1R protein levels. n=3 per group. * p <0.05, ** p <0.01. E) Representative images of western blots probed with antibodies against D1R in the overexpression experiment. F) Quantification of D1R protein levels. n=3 per group. * p <0.05, ** p <0.01. G) Representative images of western blots probed with antibodies against D1R in the silencing experiment. H) Quantification of D1R protein levels. n=3 per group. * p <0.05, ** p <0.01. I) Representative images of western blots probed with antibodies against ALP, OSX and Runx2 in the silencing experiment. J-L) Quantification of ALP, OSX and Runx2 protein levels. n=3 per group. NS: Not statistically significant, * p <0.05, ** p <0.01, vs. the control group.


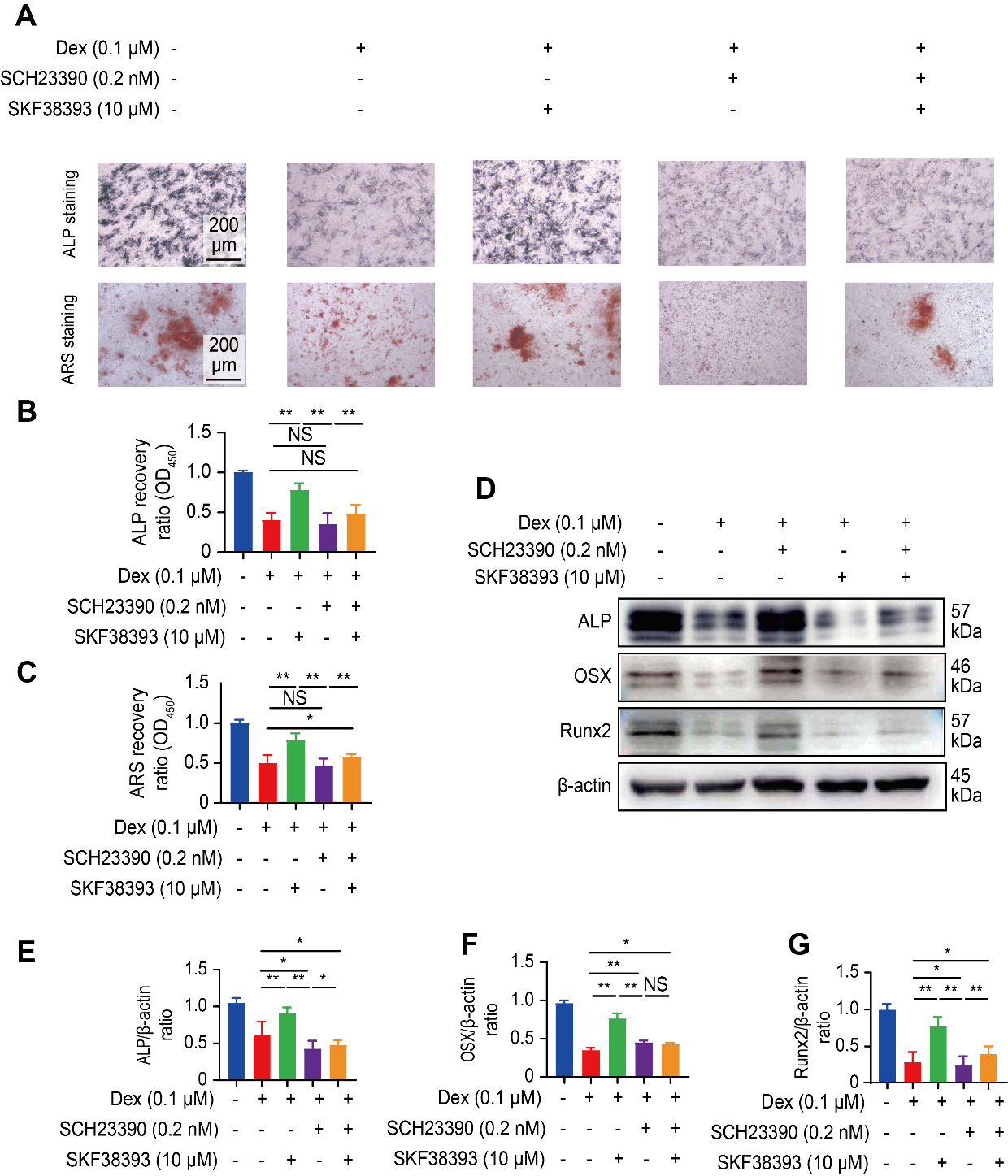


**Figure S3. Activation of D1R alleviated Dex-induced inhibition of osteoblast differentiation in Rat BMSCs cells.** A) Representative images showing ALP and ARS staining. Scale bar: 200 μm. B and C) Quantitative analysis of ALP and ARS staining. n=3 per group. NS: Not statistically significant, * p <0.05, ** p <0.01, vs. the control group. D) Representative images of western blots probed with antibodies against ALP, OSX and Runx2. E-G) Quantification of ALP, OSX and Runx2 protein levels. n=3 per group. NS: Not statistically significant, * p <0.05, ** p <0.01, vs. the control group.


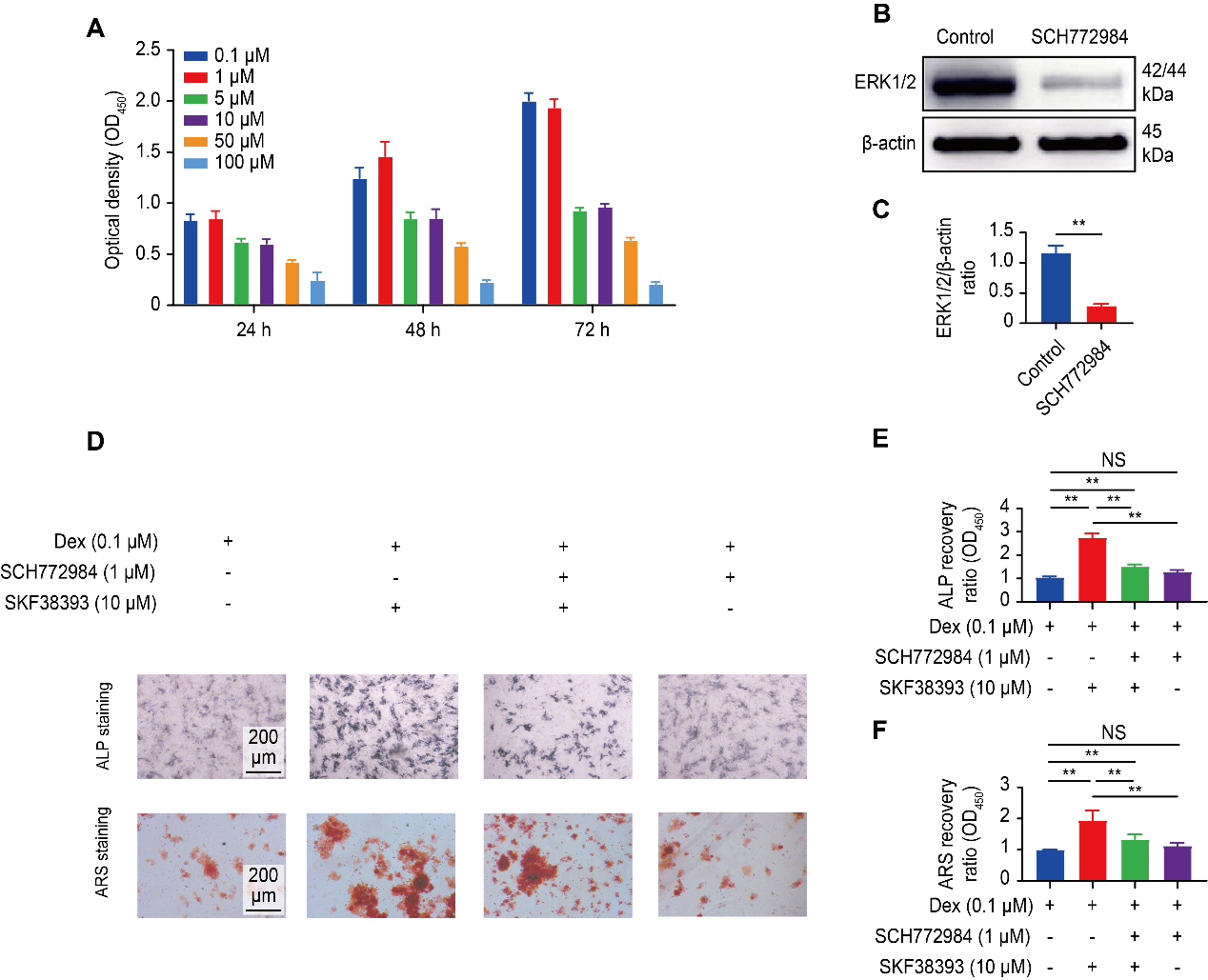


**Figure S4. ERK1/2 mediated the protective effect of D1R against Dex-mediated inhibition of osteoblast differentiation in Rat BMSCs cells.** A) CCK-8 analysis of MC3T3-E1 cells treated with SCH772984. B) Representative images of western blots probed with antibodies against ERK1/2. C) Quantification of ERK1/2 protein levels. n=3 per group. ** p <0.01. D) Representative images showing ALP and ARS staining. Scale bar: 200 μm. E and F) Quantitative analysis of ALP and ARS staining. n=3 per group. NS: Not statistically significant, * p <0.05, ** p <0.01, vs. the control group.


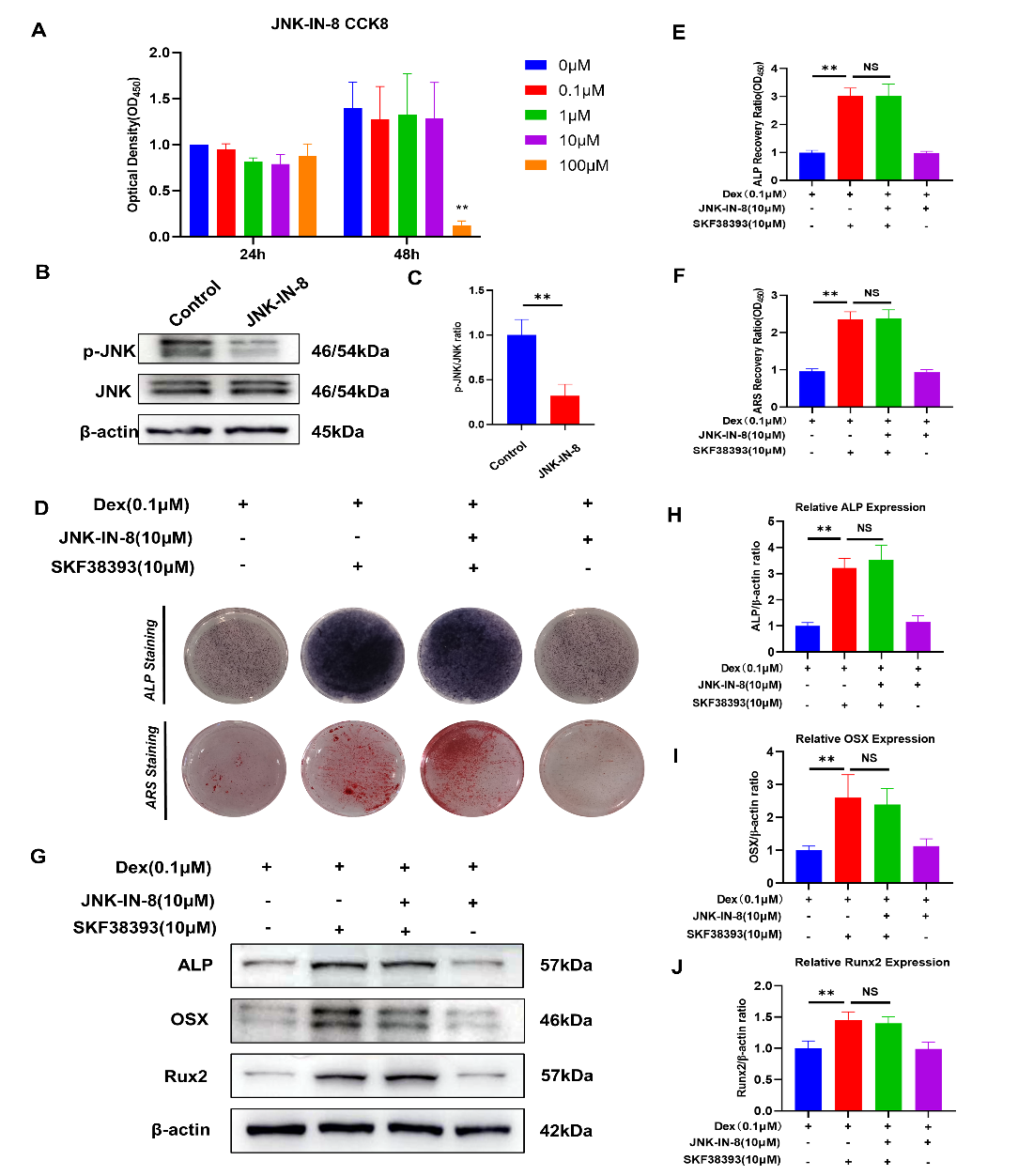


**Figure S5.** **The JNK pathway has no synergistic effects with ERK1/2 in the protective effect of activation of D1R to Dex-mediated osteoblast differentiation.** A) CCK-8 analysis of MC3T3-E1 cells treated with JNK-IN-8. B) Representative images of western blots probed with antibodies against p-JNK and JNK. C) Quantification of p-JNK/JNK. n=3 per group. ** p <0.01. D) Representative images showing ALP and ARS staining. E and F) Quantitative analysis of ALP and ARS staining. n=3 per group. NS: Not statistically significant, ** p <0.01, vs. the control group. G) Representative images of western blots probed with antibodies against ALP, OSX and Runx2. H-I) Quantification of ALP, OSX and Runx2 protein levels. n=3 per group. NS: Not statistically significant, ** p <0.01, vs. the control group.


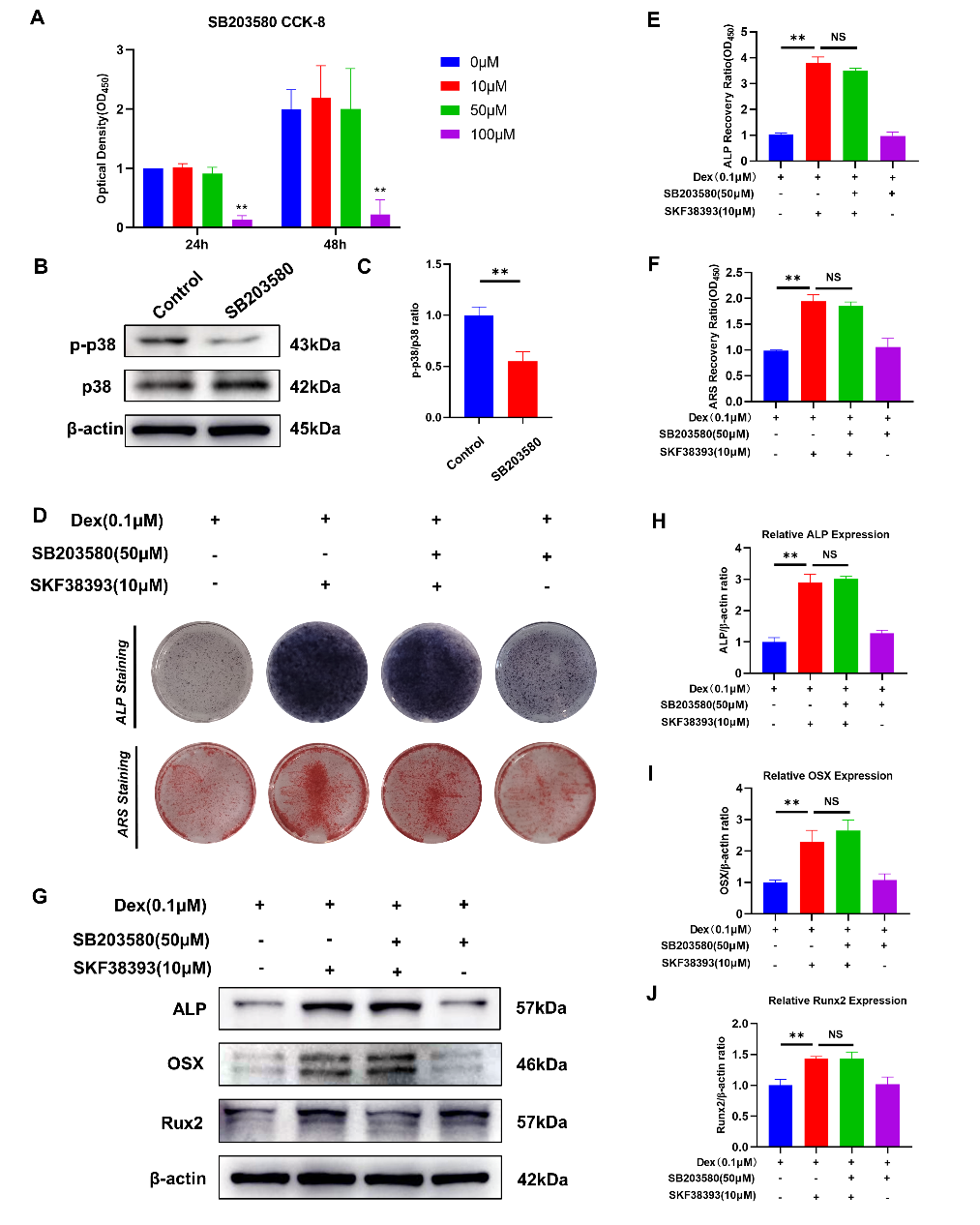


**Figure S6. The p38 pathway has no synergistic effects with ERK1/2 in the protective effect of activation of D1R to Dex-mediated osteoblast differentiation.** A) CCK-8 analysis of MC3T3-E1 cells treated with SB203580. B) Representative images of western blots probed with antibodies against p-p38 and p38. C) Quantification of p-p38/p38. n=3 per group. ** p <0.01. D) Representative images showing ALP and ARS staining. E and F) Quantitative analysis of ALP and ARS staining. n=3 per group. NS: Not statistically significant, ** p <0.01, vs. the control group. G) Representative images of western blots probed with antibodies against ALP, OSX and Runx2. H-I) Quantification of ALP, OSX and Runx2 protein levels. n=3 per group. NS: Not statistically significant, ** p <0.01, vs. the control group.


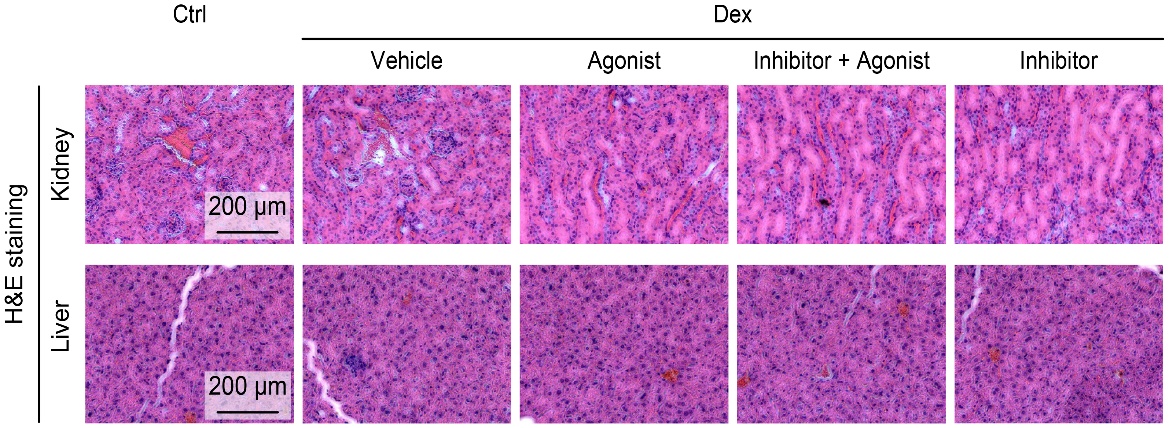


**Figure S7. H&E staining of the liver and kidney after treatment in vivo.**
